# Multi-Component Hydrogel Beads Incorporated with Reduced Graphene Oxide for pH-Responsive and Controlled Co-Delivery of Multiple Agents

**DOI:** 10.3390/pharmaceutics13030313

**Published:** 2021-02-28

**Authors:** Sreekanth Reddy Obireddy, Wing-Fu Lai

**Affiliations:** 1 Department of Chemistry, Sri Krishnadevaraya University, Ananthapuramu 515003, India; sreekanthchem7@gmail.com; 2 Department of Applied Biology and Chemical Technology, Hong Kong Polytechnic University, Hong Kong, China; 3 Ciechanover Institute of Precision and Regenerative Medicine, The Chinese University of Hong Kong (Shenzhen), Shenzhen 518172, China

**Keywords:** graphene oxide, co-delivery, controlled release, hydrogel beads, stimuli-responsiveness

## Abstract

The development of combination therapy has received great attention in recent years because of its potential to achieve higher therapeutic efficacy than that achieved by mono-drug therapy. Carriers for effective and stimuli-responsive co-delivery of multiple agents, however, are highly deficient at the moment. To address this need, this study reports the generation of multi-component hydrogel beads incorporated with reduced graphene oxide (rGO). The beads are prepared by incorporating doxorubicin (DOX)-loaded gelatine (GL) microbeads into hydrogel beads containing rGO and 5-fluorouracil (5-FU). rGO-containing beads are shown to be more effective in inhibiting the growth of MCF-7 cells via the induction of reactive oxygen species (ROS) generation. In addition, the drug release sustainability of the beads is affected by the pH of the release medium, with the release rate increasing in neutral pH but decreasing in the acidic environment. Our beads warrant further development as carriers for pH-responsive and controlled co-delivery of multiple agents.

## 1. Introduction

Recent years have witnessed an extraordinary expansion in the field of targeted drug delivery using polymeric matrices [1,2,3]. One example of polymers widely adopted is gelatine (GL), which is a biopolymer obtained by the hydrolysis of collagen. Structurally, GL contains various residues of glycine, proline and 4-hydroxy proline [4,5]. Due to its favourable properties (e.g., high biocompatibility, high biodegradability, edibility and the capacity of forming thermo-reversible gels), it has been widely used not only in food and cosmetic applications but also in drug delivery [6,7,8]. Another example is sodium alginate (SA), which is an anionic polysaccharide obtained from marine brown algae [9,10,11]. It consists of random sequences of α-L-guluronic (G) and β-D-mannuronic (M) acid residues in the polymer chain [12] and has been used extensively in food and pharmaceutical applications due to its high biocompatibility, ease of processing and gelling capacity under mild conditions [13]. In recent decades it has been used as gel-based systems for controlled, sustained and targeted drug delivery [14,15]. Besides polymers, since the turn of the last century graphene oxide (GO) has attracted extensive interest in drug delivery research partly owing to the flexibility brought about by the large number of carboxylic groups on the GO surface for subsequent functionalization [16,17,18]. GO also has a high surface area and a two-dimensional structure. This plays a vital role in the adsorption of molecules on the surface of GO sheets [19,20]. Different from GO, reduced graphene oxide (rGO) has a planar structure and this increases the drug encapsulation efficiency [21,22]. The latter has been supported by an earlier study [23], which found that the encapsulation of doxorubicin (DOX) in functionalized rGO was much higher than that in GO because of the presence of a larger number of ring structures that could form π-π stacking interactions with DOX molecules [23]. Furthermore, rGO is more biologically active than GO and hence is more effective than GO in serving as a potential drug carrier [24,25].

Despite the promise brought by advances in drug delivery as mentioned above, the efficiency of cancer therapy based on the use of chemical drugs is still unsatisfactory due to not only the low efficiency of cellular uptake and non-specific distribution in the body but also the occurrence of multiple drug resistance (MDR) [26]. An effective strategy against cancer cells requires a combination approach. Combination therapy, which refers either to the simultaneous administration of two or more pharmacologically active agents or to the combination of different types of therapy [27,28], is reported to be able to tackle infectious diseases and various types of cancer effectively, to minimize side effects and to improve prognosis [29,30,31]. Unfortunately, until now carriers for effective co-delivery of multiple agents have been highly deficient, not to mention those enabling the co-loaded agents to be released in a stimuli-responsive manner. To address this need, this study reports the generation of multi-component hydrogel beads incorporated with rGO. The beads are demonstrated to enable pH-responsive and controlled co-delivery of both DOX and 5-fluorouracil (5-FU).

## 2. Materials and Methods

### 2.1. Materials

GL, graphite powder (<20 µm) and 5-FU were obtained from Sigma–Aldrich (St. Louis, MO, USA). SA, sodium nitrate, sulphuric acid (H_2_SO_4_), potassium permanganate (KMNO_4_), 30% (v/v) hydrogen peroxide, hydrazine sulphate and calcium chloride were purchased from SdFine Chemicals (Mumbai, India). DOX was received from AspiroPharma Pvt. Ltd. (Telangana, India). Millipore water was used throughout the study.

### 2.2. Preparation of rGO

rGO was fabricated by using Hummer’s method as previously described [32]. Briefly, 3 g of graphite powder and 6 g of NaNO_2_ were added to 120 mL of H_2_SO_4_. The reaction mixture was kept in an ice bath. Upon the addition of 12 g of KMNO_4_, the reaction mixture was kept at 0–10 °C for 4 h. After that, the temperature was raised to 50 °C and constantly maintained for 4 h. The reaction mixture was cooled to 0–10 °C by using an ice bath. A total of 300 mL of water was added to it, followed by the addition of 15 mL of a 30% (*w/v*) hydrogen peroxide solution. The reaction mixture was stirred for 120 min. The product was washed with distilled water to remove acid residues, centrifuged and finally dried at 80 °C for 24 h in a hot air oven. A total of 100 mg of the product was dispersed in 100 mL of distilled water and sonicated for 1 h to get a homogeneous dispersion. Hydrazine sulphate was added to the dispersion, followed by stirring for 12 h at 60 °C to get rGO. The obtained product was washed with distilled water and dried at 80 °C for 24 h in a hot air oven.

### 2.3. Preparation of GL-DOX and GL-rGO-DOX Beads

A total of 40 mg of DOX was added to 10 mL of an 8% (*w/v*) aqueous GL solution, followed by stirring at 300 rpm until a homogeneous solution was obtained. The solution was added dropwise into 100 mL of paraffin liquid light containing 1% (v/v) Tween 80 under constant stirring at 600 rpm. After additional 20 min of stirring, 2 mL of glutaraldehyde (GA) was added. The GL-DOX beads formed were collected, filtered and treated with n-hexane to remove oil residues. They were then dried at 40 °C in a hot air oven and placed in an airtight container until further use. The same protocol was adopted to generate GL-rGO-DOX beads but during the preparation process, rGO was added to the 8% (*w/v*) aqueous GL solution along with DOX.

### 2.4. Preparation of SA-5-FU and SA-rGO-5-FU Beads

A total of 50 mg of 5-FU was added to 10 mL of a 2% (*w/v*) aqueous SA solution, followed by stirring at 300 rpm until a homogeneous solution was obtained. The solution was added dropwise into 100 mL of a 5% (*w/v*) CaCl_2_ solution. The resulting solution was kept in ambient conditions for 1 h for gelation to occur. The beads generated were collected, filtered and rinsed with distilled water. They were then air-dried and placed in an airtight container until further use. The same protocol was adopted to generateSA-rGO-5-FU beads but during the preparation process, rGO was added to the 2% (*w/v*) aqueous SA solution along with 5-FU.

### 2.5. Synthesis of SA-5-FU-rGO-GL-DOX Beads

GL-rGO-DOX microbeads were prepared as described above and were added to a 2% (*w/v*) aqueous SA solution containing 5-FU and rGO. The resulting solution was stirred at 300 rpm for 30 min. After that, it was added dropwise to 100 mL of a 5% (*w/v*) CaCl_2_ solution. The resulting solution was kept in ambient conditions for 1 h for gelation to occur. The beads generated were collected, filtered and rinsed with distilled water. They were then air-dried and placed in an airtight container until further use.

### 2.6. Structural Characterization

The structure of the samples was characterized by using a Fourier transform infrared (FTIR) spectrophotometer (Bomem MB-3000; ABB Corporate, Zurich, Switzerland) in the wavenumber range of 400–4000 cm^–1^. Differential scanning calorimetry (DSC) and thermogravimetric analysis (TGA) were performed by heating the sample at a heating rate of 10 °C/min under a nitrogen atmosphere from 35 to 600 °C. X-ray diffraction (XRD) analysis was performed by using an X-ray diffractometer (Ultima IV; Rigaku, Japan) at a scanning rate of 10°/min using Cu Kα radiation. The morphological features of the samples were studied by scanning electron microscopy (SEM) using a scanning electron microscope (JSM 840A; JEOL Ltd., Tokyo, Japan).

### 2.7. Determination of the Encapsulation Efficiency (EE)

A total of 10 mg of drug-loaded beads was dispersed in 10 mL of phosphate buffer saline (PBS) containing 0.5 mL of ethanol. The dispersion was stirred in darkness in ambient conditions for 24 h. After that, the beads were retrieved by centrifugation, crushed in PBS, sonicated for 10 min and centrifuged again. The supernatant was filtered. The drug concentration in the filtrate was determined by using a UV-Vis spectrometer at the wavelength of 478 nm for DOX and 270 nm for 5-FU. The EE was calculated by using the following formula:

$$\mathrm{EE}\;(\%)\;=\;\frac{m_{f}}{m_{d}}\times 100\%$$

where *m_f_* was the mass of the drug in the filtrate and *m_d_* was the total mass of the drug added during the preparation of the drug-loaded beads.

### 2.8. Evaluation of In Vitro Drug Release Profiles

A total of 30 mg of drug-loaded beads was put into a dialysis bag, which was then immersed in 300 mL of PBS at 37 °C at various pH values (6.8, 4.5 and 1.2). At regular time intervals, the amount of drug released from the beads was analysed by using a UV-Vis spectrometer at the wavelength of 478 nm for DOX and 270 nm for 5-FU. The release data of the drug-loaded beads were analysed by fitting them into different kinetic models (including the zero-order model, the first-order model, the Higuchi model and the Korsmeyer–Peppas model) in order to evaluate the release mechanism of the drug from the beads.

### 2.9. 3-(4,5-Dimethylthiazol-2-yl)-2,5-Diphenyl Tetrazolium Bromide (MTT) Assay

MCF-7-human breast cancer cells were purchased from the American Type Culture Collection (ATCC; Manassas, VA, USA) and were cultured in Dulbecco’s modified Eagle’s medium (DMEM) supplemented with 6% foetal calf serum (FCS) and antibiotics as previously described [33]. During the experiment, the cells were seeded in a 96-well plate at a density of 20,000 cells per well and were incubated for 24 h at 37 °C in a 5% CO_2_ atmosphere. After that, the sample was added to each well at a desired concentration, followed by incubation of the plate at 37 °C in a 5% CO_2_ atmosphere for 24 h. The MTT reagent was added to each well at a concentration of 0.5 mg/mL. After incubation at 37 °C in a 5% CO_2_ atmosphere for 3 h, the medium in each well was removed. A total of 100 μL of DMSO was added to each well. The plate was agitated on a gyratory shaker before the absorbance at 570 nm was measured by using a spectrophotometer. The IC_50_ value was determined by using the linear regression equation (viz., y = mx + c), with the value of y being set as 50 and the values of m and c being derived from the viability graph.

### 2.10. Haemolysis Assay

The haemolytic activity of the beads was determined as previously described [34]. All procedures were approved by the Ethical Committee of the Hong Kong Polytechnic University and complied with the ARRIVE guidelines and European Union Directive 2010/63/EU for animal experiments.

### 2.11. Determination of Endogenous Reactive Oxygen Species (ROS) Production

MCF-7 cells were seeded in a 6-well plate at a density of 3 × 10^5^ cells per well and were incubated for 24 h at 37 °C in a 5% CO_2_ atmosphere. After that, the sample was added to each well at a desired concentration, followed by incubation of the plate at 37 °C in a 5% CO_2_ atmosphere for 24 h. Cells were washed with PBS thrice. A total of 250 μL of trypsin-EDTA was added to each well, followed by incubation at 37 °C for 3–4 min. The cells were harvested by centrifugation at 300× *g* at 25 °C for 5 min. They were then treated with 2′,7′-dichlorodihydrofluorescein diacetate (H_2_DCFDA) (10 μM) at 37 °C for 30 min. The cells were centrifuged at 150× *g* for 5 min and washed with Dulbecco’s PBS (DPBS) to remove the extracellular dye. They were re-suspended in 400 μL of pre-warmed DPBS. The dichlorofluorescein (DCF) fluorescence of the cells was analysed by using a flow cytometer (FACSCalibur, BD Bioscience, CA, USA) at an excitation wavelength of 488 nm and an emission wavelength of 535 nm.

## 3. Results

### 3.1. Structural and Morphological Characterization

**Figure 1:**
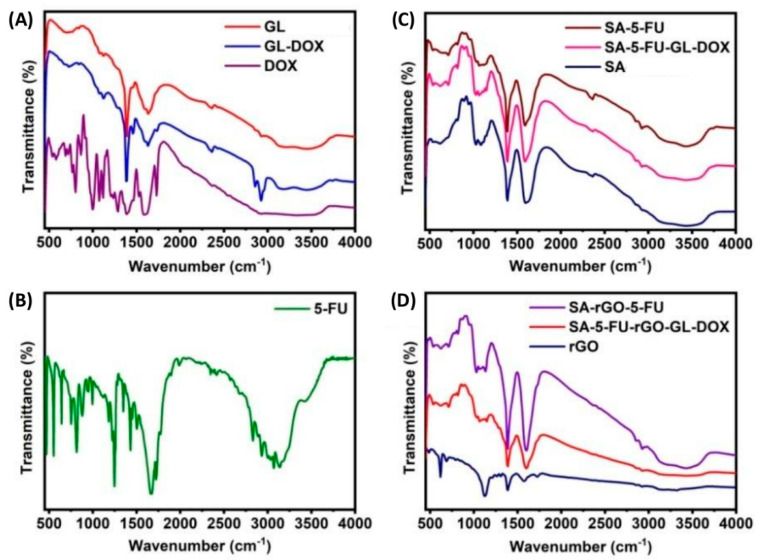
FTIR spectra of different samples: (**A**) DOX, GL-DOX and GL beads; (**B**) 5-FU; (**C**) SA-rGO-5-FU, SA-5-FU and SA beads; (**D**) rGO, SA-rGO-5-FU and SA-5-FU-rGO-GL-DOX.

DOX-GL microbeads were synthesized and embedded into an SA solution containing 5-FU and rGO for the formation of multi-component hydrogel beads. To examine possible chemical interactions among rGO, 5-FU and DOX in the beads, FTIR analysis was performed. In the FTIR spectrum of DOX, a broad peak was observed at 3327 cm^–1^ (Figure 1A). This peak was attributed to O–H and N–H stretching vibrations. The peak at 1731 cm^–1^ corresponded to C=O stretching vibrations. Peaks at 1072 cm^–1^, 1118 cm^–1^, 1388 cm^–1^ and 1589 cm^–1^ were assigned to C–O stretching vibrations of secondary alcohol groups, C–N stretching vibrations of amine groups, O–H bending vibrations of phenol and N–H bending vibrations of amine groups, respectively. The peak at 995 cm^–1^ represented the bending vibrations of C=C whereas the peak at 802 cm^–1^ was resulted from C–H bending vibrations [35]. On the other hand, the FTIR spectrum of GL beads displayed several characteristic peaks: 3345 cm^–1^ (N–H stretching vibrations), 1635 cm^–1^ (C=O stretching vibrations of amide-I), 1458 cm^–1^ (C=O stretching vibrations of amide-II) and 1380 cm^–1^ (C–N stretching vibrations). After being loaded with DOX, the peak assigned to the stretching vibrations of C=O was shifted to 1627cm^–1^ due to the formation of hydrogen bonding interactions between the hydroxyl group of DOX and the NH_2_ group of GL. A new peak was also observed at 1735 cm^–1^ due to the presence of DOX in the matrix of the GL beads. These results confirmed that DOX was successfully loaded into GL microbeads.

The FTIR spectrum of 5–FU showed peaks at 2831–3138 cm^–1^ due to N–H and C–H stretching vibrations whereas the peaks at 1662 cm^–1^ and 1248 cm^–1^ were contributed by C=O stretching vibrations and C–F stretching vibrations, respectively (Figure 1B). The FTIR spectrum of SA beads showed the following characteristic peaks: 3417 cm^–1^ (O–H stretching vibrations), 1601 cm^–1^ (C=O stretching vibrations) and 1388 cm^–1^ (C–O stretching vibrations) (Figure 1C). After being loaded with 5-FU, the signal assigned to the stretching vibrations of C=O was shifted to 1589 cm^–1^ due to the occurrence of electrostatic interactions between 5-FU (–NH group) and SA (–OH group). New peaks were also noted at 711cm^–1^ and 1149 cm^–1^ due to stretching and bending vibrations of C–F. These findings confirmed that 5-FU was successfully loaded into the polymer matrix.

The FTIR spectrum of rGO showed characteristic peaks at 3317 cm^–1^ and 1728 cm^–1^. These peaks were attributed to stretching vibrations of O–H and C=O groups, respectively (Figure 1D). The peaks at 1573 cm^–1^, 1388 cm^–1^ and 1126 cm^–1^ could also be assigned to the stretching vibrations of C=C, C–O and C–O–C groups, respectively. After the incorporation of rGO into the hydrogel matrix formed by SA, the peak at 1728 cm^–1^ disappeared. Meanwhile, the peak (1601 cm^–1^) assigned to C=O stretching vibrations in the spectrum of SA beads was shifted to 1596 cm^–1^ in the spectra of both SA-rGO-5-FU and SA-5-FU-rGO-GL-DOX due to the interactions between rGO and the SA matrix and those between rGO (C=O group) and 5-FU (–NH group). This observation was in good agreement with that reported by Piao and Chen [36] who noted that the C=O stretching vibrations of rGO disappeared upon interactions between the C=O group of rGO and the amine group of a polymer matrix. In addition, a new peak appeared at ~871 cm^–1^ in the spectra of both SA-rGO-5-FU and SA-5-FU-rGO-GL-DOX due to π-π stacking interactions between rGO and DOX/5-FU. This statement was consistent with the observation made by Ma and co-workers [37] who reported that DOX interacted with rGO via strong π-π stacking interactions. Our results evidenced that both rGO and 5-FU were loaded into the polymer matrix.

**Figure 2:**
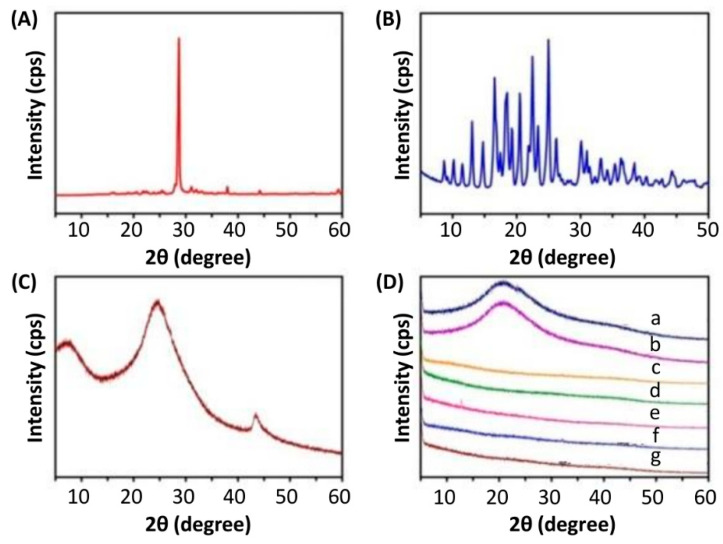
XRD spectra of (**A**) 5-FU, (**B**) DOX, (**C**) rGO and (**D**) different beads: (a) GL beads; (b) GL-DOX; (c) SA beads; (d) SA-5-FU; (e) SA-5-FU-GL-DOX; (f) SA-rGO-5-FU; and (g) SA-5-FU-rGO-GL-DOX.

Apart from using FTIR, the structure of the beads was characterized by using XRD (Figure 2). The XRD pattern of 5-FU showed a peak at 28.68° whereas that of DOX showed multiple peaks between 13° and 25°. This indicated the crystalline nature of 5-FU and DOX. These peaks were not found in drug-loaded beads, suggesting the conversion of drug molecules from the crystalline state into an amorphous state. In addition, the XRD pattern of rGO showed a broad peak at around 20–30°. This confirmed the formation of rGO from graphite [38] and was in good agreement with that reported by Ma and co-workers [37] who also observed a broad peak at around 20–30° in the XRD pattern of rGO fabricated by using the sodium salt of riboflavin-5′-phosphate as both a reducing reagent and a stabilizer. The diffraction peak of rGO, however, was absent in the XRD patterns of both SA-rGO-5-FU and SA-5-FU-rGO-GL-DOX, indicating that rGO lost its crystal nature in the hydrogel matrix and was dispersed as nanosheets in the hydrogel beads.

**Figure 3:**
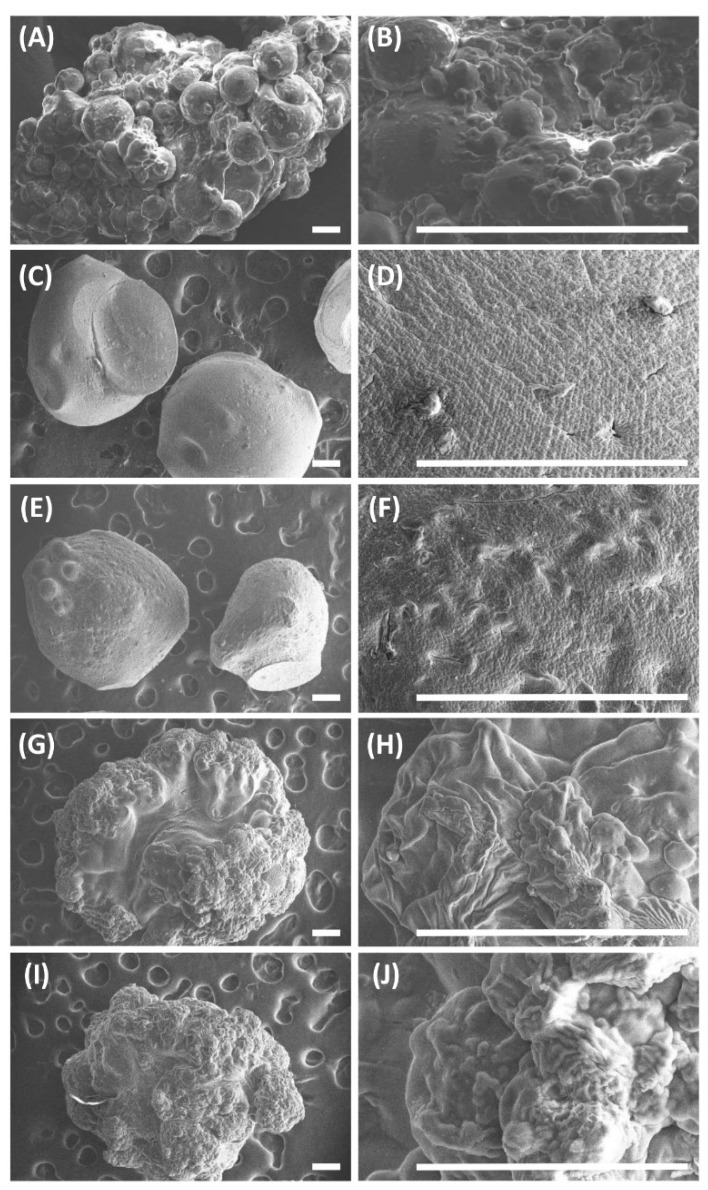
SEM images of (**A**,**B**) GL-DOX, (**C**,**D**) SA-5-FU, (**E**,**F**) SA-rGO-5-FU, (**G**,**H**) SA-5-FU-GL-DOX and (**I**,**J**) SA-5-FU-rGO-GL-DOX. Scale bar = 200 μm.

The morphological features of the beads were studied using SEM (Figure 3). All of the beads were spherical and wrinkled and had a rough outer layer. The outer surface of SA-rGO-5-FU was rougher than that of SA-5-FU due to the presence of rGO. In addition, the outer surface of SA-5-FU-GL-DOX and SA-5-FU-rGO-GL-DOX was the roughest among all beads examined and displayed visible wrinkles. This was largely due to the presence of GL beads in the polymer matrix of the multi-component hydrogel beads.

### 3.2. Thermal Analysis

**Figure 4:**
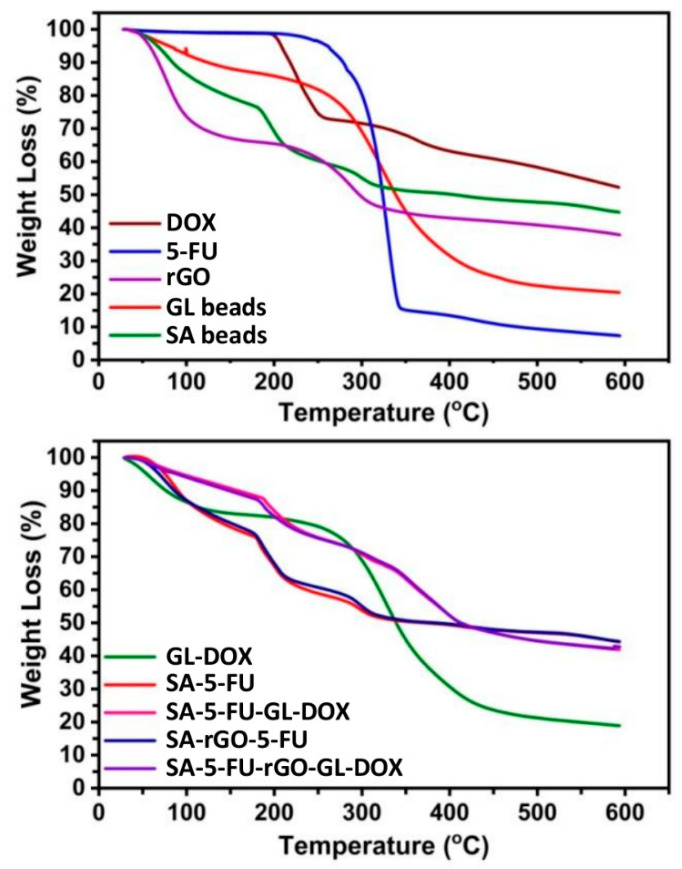
TGA curves of different beads (GL beads, SA beads, GL-DOX, SA-5-FU, SA-5-FU-GL-DOX, SA-rGO-5-FU and SA-5-FU-rGO-GL-DOX) and their constituents (rGO, DOX and 5-FU).

**Figure 5:**
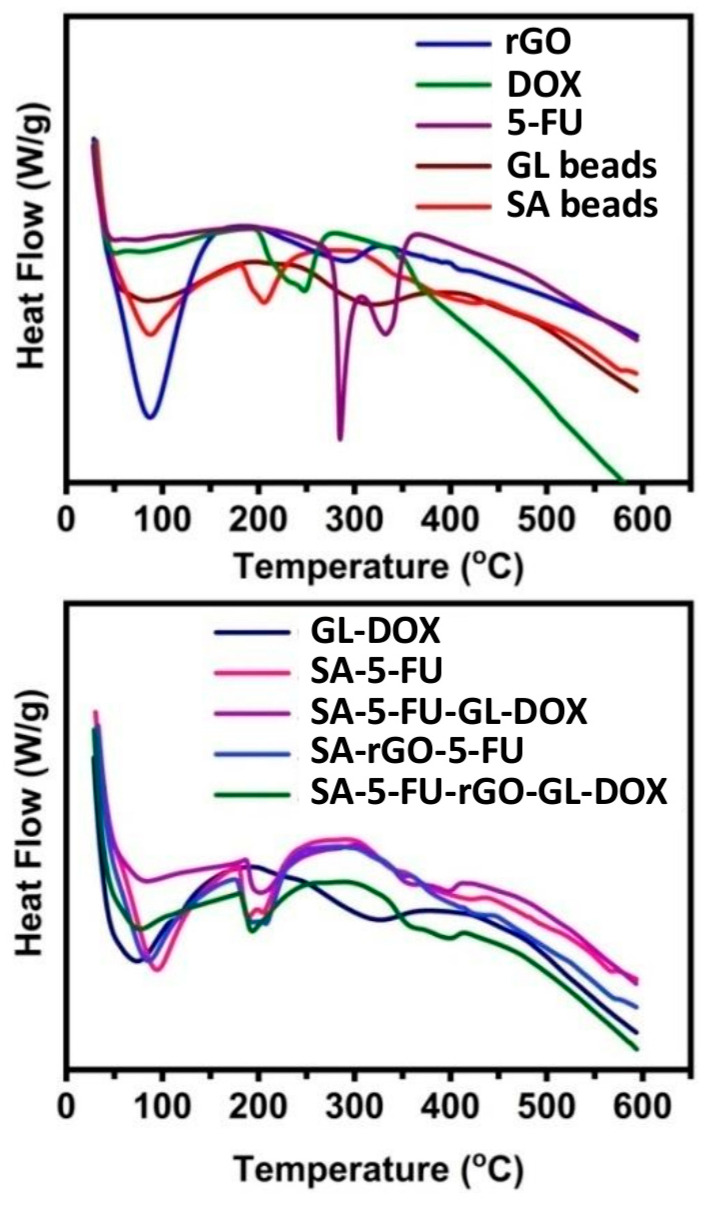
DSC curves of different beads (GL beads, SA beads, GL-DOX, SA-5-FU, SA-5-FU-GL-DOX, SA-rGO-5-FU and SA-5-FU-rGO-GL-DOX) and their constituents (rGO, DOX and 5-FU).

The thermal stability of the beads and their constituents was analysed by using TGA and DSC (Figure 4 and Figure 5). The TGA curve of rGO showed two weight loss steps. The first step occurred between 35 °C and 151 °C with a weight loss of 27%. This step was due to the evaporation of adsorbed moisture on the sample surface. The second step occurred from 215 °C to 600 °C with a weight loss of 36% and was attributed to the pyrolysis of oxygen-containing functional groups [39]. The TGA curves of 5-FU and DOX revealed that both drugs were thermally stable up to around 190 °C before degradation occurred. At 600 °C, the residual masses of SA beads, GL beads, SA-5-FU, GL-DOX, SA-rGO-5-FU, SA-5-FU-GL-DOX and SA-5-FU-rGO-GL-DOX were 44%, 20%, 43%, 18%, 44%, 43% and 43%, respectively. These findings showed that the drug-loaded rGO-containing beads had good thermal stability. In the DSC curve of 5-FU, a sharp peak was observed at 285 °C whereas in that of DOX, a peak at 207 °C was found. These peaks indicated the melting point of the drug. They were absent in the DSC curves of drug-loaded beads, suggesting that the drug molecules were dispersed in the hydrogel matrix at the molecular level.

### 3.3. Drug Encapsulation and pH-Responsive Release

**Figure 6:**
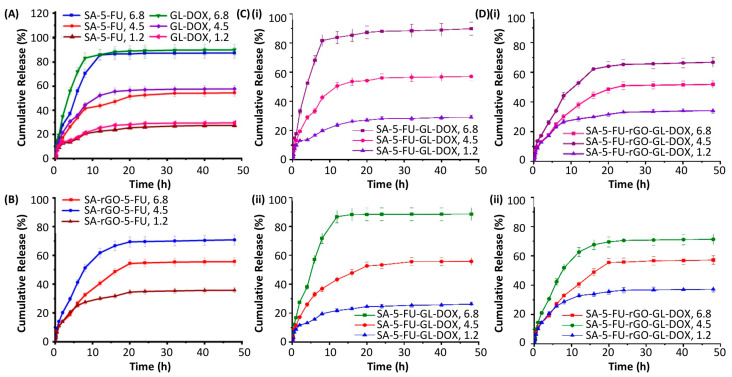
Release profiles of the loaded drug from (**A**) GL-DOX and SA-5-FU and from (**B**) SA-rGO-5-FU in the release medium at different pH values (6.8, 4.5 and 1.2). (**C**) Release profiles of (**i**) DOX and (**ii**) 5-FU from SA-5-FU-GL-DOX in the release medium at different pH values (6.8, 4.5 and 1.2). (**D**) Release profiles of (**i**) DOX and (**ii**) 5-FU from SA-5-FU-rGO-GL-DOX in the release medium at different pH values (6.8, 4.5 and 1.2).

The EE of rGO-containing beads (SA-rGO-5-FU and SA-5-FU-rGO-GL-DOX) was much higher than that of the beads in which rGO was absent (Table 1). This was attributed partly to the π-π stacking interactions and hydrogen bonding interactions between rGO and the drug (DOX and 5-FU). The release profiles of the drug-loaded beads were examined at 37 °C in PBS at various pH values (6.8, 4.5 and 1.2) (Figure 6). At pH 6.8, approximately 90% of the loaded drug could be successfully released from SA-5-FU, GL-DOX and SA-5-FU-GL-DOX but at pH 4.5, this percentage dropped to around 50–60%. After the incorporation of rGO, the release rate of the beads (SA-rGO-5-FU and SA-5-FU-rGO-GL-DOX) at pH 4.5 was higher than that at pH 6.8. This was explained by changes in the formation of hydrogen bonding interactions between the functional groups of rGO (–COOH and –OH) and those of the drug (–NH and –OH). At pH 4.5, H^+^ ions in the release medium disrupted the hydrogen bonding interactions between rGO and the drug, leading to a higher percentage of the loaded drug being released; whereas at pH 6.8, the formation of hydrogen bonding interactions between rGO and the drug was not disrupted and this reduced the amount of the drug successfully released from the beads [40]. At pH 1.2, the percentage of drug release was lower than that at pH 4.5. This was explained by the limited swelling capacity of ionically crosslinked alginate-based hydrogel beads in such an acidic environment as reported by earlier studies [41,42]. Such pH-responsiveness is highly favourable for oral drug administration because it enables the loaded drug to be protected from the acidic gastric environment and allows the drug molecules to be released when the carrier gets to the intestinal region.

The kinetics of drug release was determined by fitting the release data into various kinetic models (including the zero-order model, the first-order model, the Higuchi model and the Korsmeyer–Peppas model) (Table 2). Based on the regression coefficient (r^2^) values, the release profiles of all drug-loaded beads (GL-DOX, SA-5-FU, SA-5-FU-GL-DOX, SA-rGO-5-FU and SA-5-FU-rGO-GL-DOX) fitted the Higuchi model, suggesting that the drug release process involved the penetration of the release medium into the hydrogel matrix for the loaded drug molecules to diffuse through the pores into the external environment. The release data were fitted into the Korsmeyer–Peppas equation:

$$\;\frac{M_{t}}{M_{\propto }}\;=\;{kt}^{n}$$

where *M_t_* is the mass percentage of the loaded drug released at time *t*, *M_α_* is the total amount of the drug loaded into the beads, *k* is the release rate constant and *n* is the diffusion exponent. The diffusion exponent values were in the range of 0.402–0.665. This suggested that the release process was mediated by the anomalous or non-Fickian type diffusion.

### 3.4. Evaluation of Haemolytic and Anti-Cancer Properties

**Figure 7:**
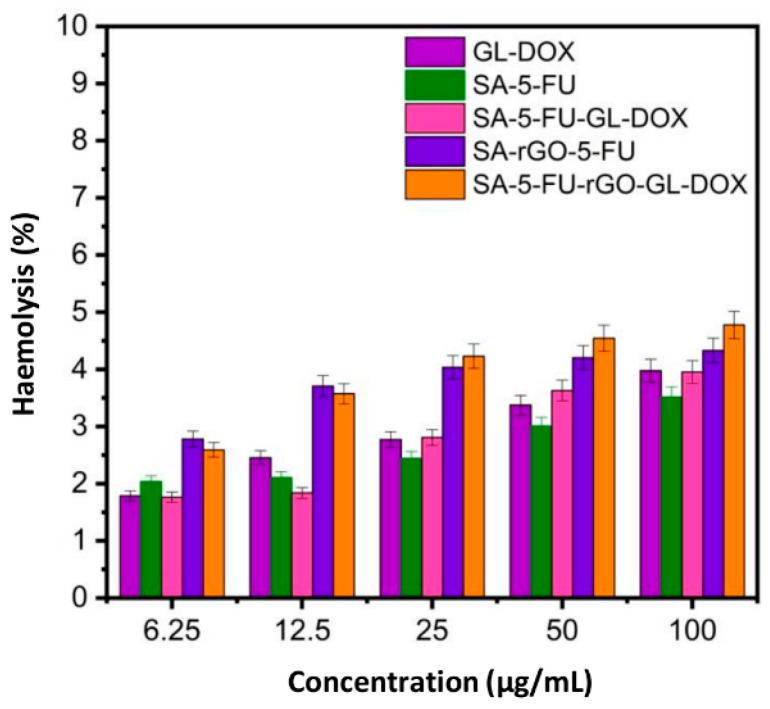
Percentages of erythrocytes lysed upon treatment with different beads (GL-DOX, SA-5-FU, SA-5-FU-GL-DOX, SA-rGO-5-FU and SA-5-FU-rGO-GL-DOX) at different concentrations.

Haemolysis is an important factor determining the biocompatibility of a drug carrier. The treatment of erythrocytes with GL-DOX and SA-5-FU-GL-DOX led to a low percentage of haemolysis (Figure 7). Although the incorporation of rGO into the beads caused an increase in the percentage of haemolysis, the overall percentage was still less than 5%. This demonstrated that the rGO-containing beads were safe for drug delivery applications.

**Figure 8:**
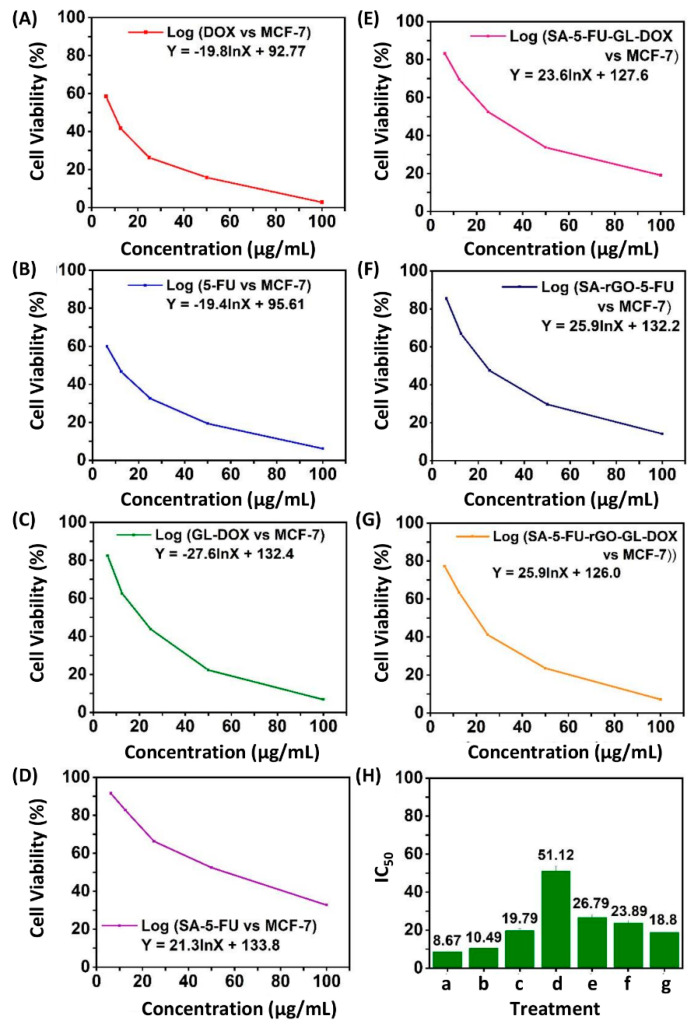
The viability of MCF-7 cells upon treatment with (**A**) DOX, (**B**) 5-FU, (**C**) GL-DOX, (**D**) SA-5-FU, (**E**) SA-5-FU- GL-DOX, (**F**) SA-rGO-5-FU and (**G**) SA-5-FU-rGO-GL-DOX. (**H**) The inhibitory concentration 50 (IC_50_) values of (a) DOX, (b) 5-FU, (c) GL-DOX, (d) SA-5-FU, (e) SA-5-FU-GL-DOX, (f) SA-rGO-5-FU and (g) SA-5-FU-rGO-GL-DOX.

**Figure 9:**
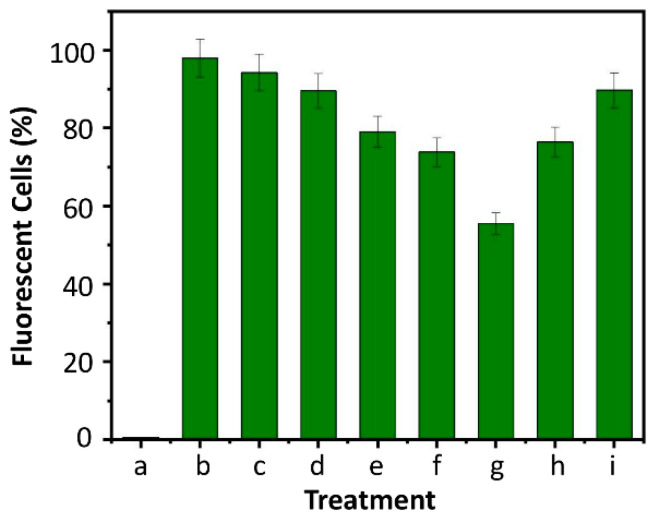
Percentages of cells displaying dichlorofluorescein (DCF) fluorescence after (a) no treatment or (b–i) treatment with various agents: (b) H_2_O_2_; (c) DOX; (d) 5-FU; (e) GL-DOX; (f) SA-5-FU; (g) SA-5-FU-GL-DOX; (h) SA-rGO-5-FU and (i) SA-5-FU-rGO-GL-DOX.

The anti-cancer capacity of 5-FU, DOX, GL-DOX, SA-5-FU, SA-5-FU-GL-DOX, SA-rGO-5-FU and SA-5-FU-rGO-GL-DOX were examined in MCF-7 cells (Figure 8). The MTT results showed that beads containing both DOX and 5-FU could kill more cancer cells than those containing either of the drugs and that the anti-cancer effect increased in a dose-dependent manner. This was in good agreement with that reported by Zhang and co-workers [43] who co-delivered 5-fluorodeoxyuridine and DOX using gold nanoparticles and showed that combination therapy led to higher anti-tumour activity than mono-drug therapy. Compared with SA-5-FU-GL-DOX, SA-5-FU-rGO-GL-DOX displayed stronger anti-cancer effects because of its higher drug content due to its higher EE, though the intrinsic anti-cancer property of rGO also played a role. After treatment with the drug-loaded beads, MCF-7 cells were stained with H_2_DCFDA. The cell treatment was found to increase the endogenous ROS level in MCF-7 cells (Figure 9), indicating that the anti-cancer effect of the drug-loaded beads was at least partially mediated by the induction of ROS generation.

## 4. Conclusions

The development of carriers for co-delivery of multiple agents can streamline the implementation of combination therapy and hence has a high practical value. This study reported the generation and characterization of multi-component rGO-containing hydrogel beads into which 5-FU and DOX could be co-loaded. The presence of rGO not only increased the EE and thermal stability of the beads but also changed the release profiles and anti-cancer capacity. Importantly, our beads showed pH-responsive drug release behaviour. Among different pH values (6.8, 4.5 and 1.2) examined, the percentage of drug release was the lowest at pH 1.2. Such release behaviour is highly favourable for oral drug administration because it enables the loaded drug to be protected from the acidic gastric environment and allows the drug molecules to be released when the carrier reaches the intestinal region. Along with their high biocompatibility, as demonstrated by the haemolysis assay, our multi-component beads are promising carriers showing high potential for exploitation for future use in co-delivery of multiple agents.

## Figures and Tables

**Table 1:** The encapsulation efficiency (EE) of different beads.

Bead	EE (%)	
	5-FU	DOX
SA-5-FU	62.73	N/A
GL-DOX	N/A	69.55
SA-rGO-5-FU	73.38	N/A
SA-5-FU-GL-DOX	61.04	67.48
SA-5-FU-rGO-GL-DOX	71.62	73.15

Abbreviation: N/A, not applicable.

**Table 2:** Release kinetic parameters of different drug-loaded beads at pH 6.8, 4.5 and 1.2.

Bead	Drug	pH	Korsmeyer–Peppas		Zero-order		First-order		Higuchi	
			n	r^2^	K_0_	r^2^	K_1_	r^2^	K_H_	r^2^
SA-5-FU	5-FU	6.8	0.993	0.660	0.586	2.786	0.674	0.136	0.821	16.83
		4.5	0.986	0.538	0.663	1.681	0.730	0.031	0.882	10.08
		1.2	0.949	0.411	0.666	0.845	0.696	0.011	0.882	5.11
GL-DOX	DOX	6.8	0.975	0.531	0.515	2.899	0.651	0.203	0.765	17.80
		4.5	0.994	0.538	0.612	1.821	0.669	0.038	0.846	11.02
		1.2	0.974	0.406	0.651	0.923	0.679	0.012	0.871	05.60
SA-rGO-5-FU	5-FU	6.8	0.981	0.584	0.738	1.692	0.784	0.030	0.919	9.961
		4.5	0.988	0.630	0.647	2.204	0.725	0.060	0.868	13.19
		1.2	0.974	0.468	0.618	1.116	0.658	0.016	0.851	06.77
SA-5-FU-GL-DOX	5-FU	6.8	0.996	0.665	0.583	2.818	0.669	0.141	0.818	17.03
		4.5	0.985	0.537	0.686	1.708	0.755	0.032	0.897	10.21
		1.2	0.951	0.413	0.660	0.803	0.687	0.010	0.877	04.86
	DOX	6.8	0.972	0.545	0.540	0.285	0.690	0.181	0.784	17.41
		4.5	0.994	0.556	0.637	1.777	0.697	0.035	0.863	10.71
		1.2	0.947	0.402	0.683	0.890	0.712	0.011	0.891	05.37
SA-5-FU-rGO-GL-DOX	5-FU	6.8	0.985	0.570	0.748	1.725	0.797	0.031	0.925	10.15
		4.5	0.986	0.617	0.643	2.226	0.722	0.063	0.866	13.35
		1.2	0.977	0.486	0.621	1.158	0.662	0.017	0.854	07.01
	DOX	6.8	0.985	0.583	0.741	1.572	0.789	0.026	0.921	9.254
		4.5	0.981	0.604	0.695	2.047	0.764	0.047	0.898	12.15
		1.2	0.981	0.509	0.633	1.054	0.670	0.014	0.861	6.370

## Data Availability

All data generated or analysed during this study are included in this published article.
